# Extracorporeal carbon dioxide removal requirements for ultraprotective mechanical ventilation: Mathematical model predictions

**DOI:** 10.1111/aor.13601

**Published:** 2019-12-15

**Authors:** John Kenneth Leypoldt, Jacques Goldstein, Dominique Pouchoulin, Kai Harenski

**Affiliations:** ^1^ Polish Academy of Sciences Nalecz Institute of Biocybernetics and Biomedical Engineering Warsaw Poland; ^2^ Baxter World Trade SPRL Braine l'Alleud Belgium; ^3^ Gambro Industries Meyzieu France; ^4^ Baxter Deutschland GmbH Unterschleissheim Germany

**Keywords:** carbon dioxide removal, extracorporeal, mathematical model, mechanical ventilation, physiological simulation

## Abstract

Extracorporeal carbon dioxide (CO_2_) removal (ECCO_2_R) facilitates the use of low tidal volumes during protective or ultraprotective mechanical ventilation when managing patients with acute respiratory distress syndrome (ARDS); however, the rate of ECCO_2_R required to avoid hypercapnia remains unclear. We calculated ECCO_2_R rate requirements to maintain arterial partial pressure of CO_2_ (PaCO_2_) at clinically desirable levels in mechanically ventilated ARDS patients using a six‐compartment mathematical model of CO_2_ and oxygen (O_2_) biochemistry and whole‐body transport with the inclusion of an ECCO_2_R device for extracorporeal veno‐venous removal of CO_2_. The model assumes steady state conditions. Model compartments were lung capillary blood, arterial blood, venous blood, post‐ECCO_2_R venous blood, interstitial fluid and tissue cells, with CO_2_ and O_2_ distribution within each compartment; biochemistry included equilibrium among bicarbonate and non‐bicarbonate buffers and CO_2_ and O_2_ binding to hemoglobin to elucidate Bohr and Haldane effects. O_2_ consumption and CO_2_ production rates were assumed proportional to predicted body weight (PBW) and adjusted to achieve reported arterial partial pressure of O_2_ and a PaCO_2_ level of 46 mmHg at a tidal volume of 7.6 mL/kg PBW in the absence of an ECCO_2_R device based on average data from LUNG SAFE. Model calculations showed that ECCO_2_R rates required to achieve mild permissive hypercapnia (PaCO_2_ of 46 mmHg) at a ventilation frequency or respiratory rate of 20.8/min during mechanical ventilation increased when tidal volumes decreased from 7.6 to 3 mL/kg PBW. Higher ECCO2R rates were required to achieve normocapnia (PaCO2 of 40 mmHg). Model calculations also showed that required ECCO2R rates were lower when ventilation frequencies were increased from 20.8/min to 26/min. The current mathematical model predicts that ECCO2R rates resulting in clinically desirable PaCO2 levels at tidal volumes of 5‐6 mL/kg PBW can likely be achieved in mechanically ventilated ARDS patients with current technologies; use of ultraprotective tidal volumes (3‐4 mL/kg PBW) may be challenging unless high mechanical ventilation frequencies are used.

## 1 INTRODUCTION

When managing patients with acute respiratory distress syndrome (ARDS), it is strongly recommended to target tidal volumes of 6 mL/kg predicted body weight (PBW)^1^ to limit overdistention of lung tissues and ventilator‐induced lung injury (VILI).^2^ Such low tidal volumes may however result in hypercapnia and respiratory acidosis, potentially limiting their routine use. Several clinical strategies for minimizing hypercapnia and VILI include higher levels of positive end‐expiratory pressure, higher ventilation frequencies or respiratory rates, and the use of prone positioning or extracorporeal carbon dioxide (CO_2_) removal (ECCO_2_R).^3^, ^4^ The degree to which hypercapnia can be mitigated with such strategies, thereby improving patient outcomes, remains to be demonstrated in robust clinical trials.

LUNG SAFE^5^ was an international observational cohort study to evaluate the incidence and outcomes of ARDS and to assess practice patterns when treating ARDS patients. The results from that study suggest that clinicians favor a balance of mild permissive hypercapnia (as defined in^6^) and moderate tidal volumes; the mean tidal volume in LUNG SAFE was 7.6 mL/kg PBW. However, several small clinical studies^7^, ^8^, ^9^, ^10^, ^11^, ^12^ have suggested that the use of tidal volumes lower than 6 mL/kg PBW, so‐called ultraprotective ventilation strategies, may provide additional clinical benefits. Because the achievement of such ultralow tidal volumes will exacerbate hypercapnia, ARDS patients so treated will likely require the use of a combination of clinical strategies that include ECCO_2_R.

There are several ECCO_2_R devices currently available^13^ with varying abilities to remove CO_2_ depending on the rate of blood flow through the device and the surface area available for transmembrane diffusion. These devices have generally been categorized as lower or higher CO_2_ extraction devices^14^, ^15^ with the former containing relatively small membrane surface area using blood flow rates ≤500 mL/min and the latter containing relatively large membrane surface area using blood flow rates >500 mL/min. Improvement in ECCO_2_R technologies has great potential as such devices can be integrated into a continuous renal replacement extracorporeal circuit^13^ and their efficacy for CO_2_ removal may be increased by using physical principles other than diffusion such as electrodialysis^13^ or novel membrane fabrication techniques.^16^, ^17^ However, the ECCO_2_R requirements for achieving adequate blood gas chemistry or normocapnia in lung‐protective strategies have not been quantitatively evaluated except in preclinical studies.^18^

In this report, we explore the effects of low tidal volumes and ECCO_2_R on total body CO_2_ content and blood gas chemistry using a mathematical model of CO_2_ biochemistry and transport. In so doing, this study defines the ECCO_2_R requirements for extracorporeal devices to achieve adequate blood gas chemistry when using protective and ultraprotective strategies during mechanical ventilation in ARDS patients.

## 2 METHODS

The six‐compartment model of CO_2_ and oxygen (O_2_) whole‐body storage and transport employed in this study was that developed by others,^19^ only modified structurally by the inclusion of an ECCO_2_R device for extracorporeal veno‐venous removal of CO_2_. Only steady state conditions were considered. The compartments included were: lung capillary blood, arterial blood, venous blood, post‐ECCO_2_R venous blood, interstitial fluid, and tissue cells; the model separately calculated the acid‐base and O_2_ contents of each compartment. As proposed previously,^19^ it was assumed that interstitial fluid and tissue cell acid‐base and O_2_ contents could be calculated from those in venous blood only; thus, those compartments were not directly involved in formulating mass transport relationships. The volumes of each fluid compartment were assumed as 0.75% (arterial blood), 6.75% (venous blood), 0% (lung capillary blood and post‐ECCO_2_R venous blood), 12.6% (interstitial fluid), and 20% (tissue cells) of the predicted body weight. The latter volume was based on assuming muscle is the only store of CO_2_ from a well‐perfused tissue. Muscle tissue volume is an underestimate of total tissue volume containing CO_2_ and neglects the largest store in bone; it has been previously proposed to be an approximation for estimating changes in CO_2_ tissue content that occur over short periods of time.^19^

**Figure 1:**
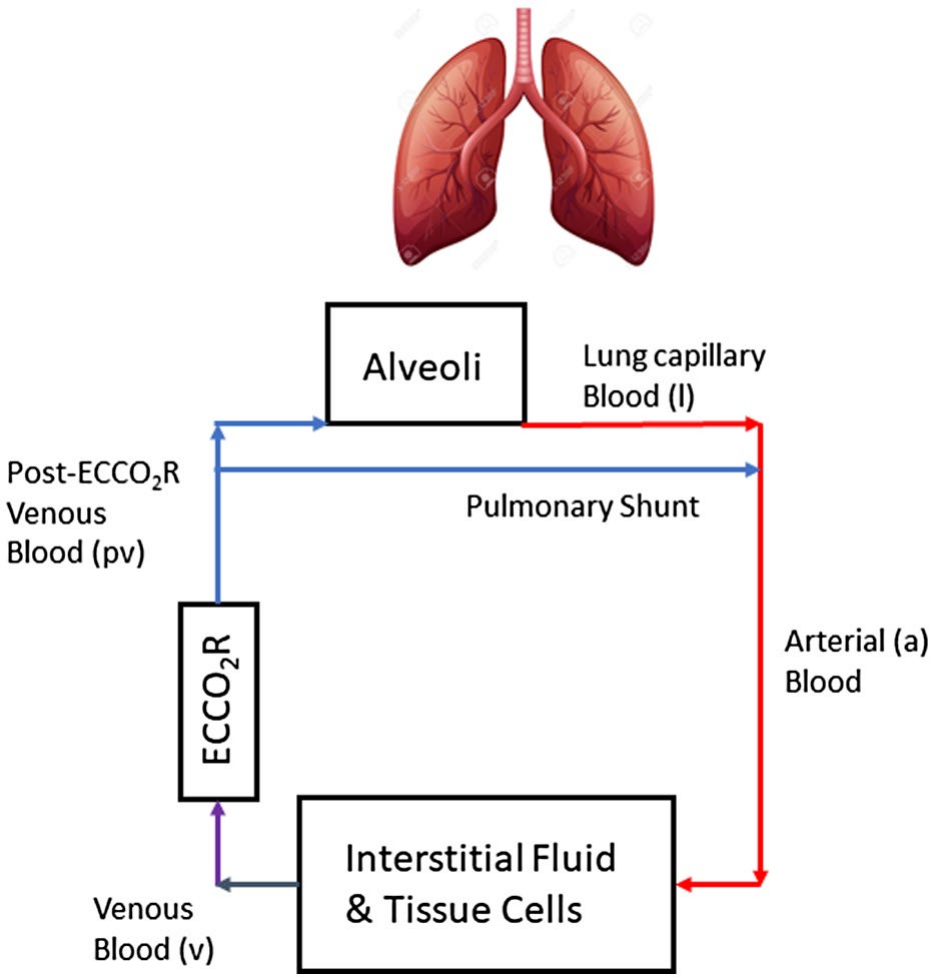
A schematic of the compartmental model for whole‐body CO_2_ transport used in this study. Blood from four compartments are involved in the CO_2_ whole‐body transport model as detailed in the Appendix. The ECCO_2_R device was added to the model previously described by others^19^ [Color figure can be viewed at https://www.wileyonlinelibrary.com]

Figure 1 shows a schematic of the mathematical model for total CO_2_ whole‐body transport. The model for O_2_ whole‐body transport is identical to that proposed previously^19^ as O_2_ transport across the ECCO_2_R device was neglected. The lung was simply characterized by three separate elements: alveoli that are involved in gas exchange describing ventilation and perfusion of the lung, alveoli dead space (ventilation but no perfusion), and a pulmonary shunt (perfusion but no ventilation). The lung was considered to be mechanically ventilated with a given frequency or respiratory rate, a tidal volume per PBW and a ratio of dead space to tidal volume fixed at 0.60; the latter is the average value reported for patients with ARDS.^20^ This lung model allows the volume of gases flowing into the alveoli per minute to be calculated from the fractional concentration of these gases in inspired and expired air. The partial pressures of CO_2_ and O_2_ in the alveoli and lung capillary blood were assumed in equilibrium, that is, no resistance to gas exchange across the alveoli/lung capillary membrane. Total concentrations of CO_2_ and O_2_ in arterial blood were the weighted average of their concentrations in lung capillary and pulmonary shunt blood flows. The ECCO_2_R device was characterized by a CO_2_ removal rate, expressed as mL of CO_2_ removed per min. The equations describing CO_2_ whole‐body transport are outlined in the Appendix; the complete model equations describing O_2_ whole‐body transport and other elements of the model were previously described.^19^

Acid‐base and O_2_ chemistry of blood, including separate chemical reactions in plasma and erythrocytes, were formulated as described previously.^21^, ^22^ The acid‐base reactions included equilibrium for bicarbonate and nonbicarbonate buffers in both plasma and erythrocytes and the binding of CO_2_ to hemoglobin in the erythrocytes. The model included competitive binding of O_2_, CO_2_, and hydrogen ions to hemoglobin to elucidate Bohr and Haldane effects as described by others.^21^ This model of acid‐base and O_2_ chemistry in blood is comprehensive; it requires solving, in general, 28 equations and 12 parameters for each compartment containing blood (lung capillary blood, arterial blood, venous blood, and post‐ECCO_2_R venous blood). This model has been previously shown to accurately describe changes in acid‐base chemistry after addition to or removal of CO_2_ and strong acid from blood,^21^ the mixing of blood samples with differing contents of CO_2_ and O_2_,^23^ and the distribution of bicarbonate to interstitial fluids.^19^ The chemical mass action equations are not described here as they can be found in detail elsewhere.^21^ The gas solubility parameters and equilibrium constants for the various acid‐base reactions in blood were those reported previously.^21^ The governing equations describing whole‐body biochemistry and transport as outlined above and in the Appendix were solved using Matlab R2018a (Mathworks, Natick, MA, USA).

The total CO_2_ concentrations of arterial blood, venous blood, and post‐ECCO_2_R venous blood were calculated as the volume‐weighted average of the CO_2_ contents in plasma and erythrocytes based on the calculated partial pressure of CO_2_ (PaCO_2_), bicarbonate concentration, and carbaminohemoglobin concentrations, the latter in erythrocytes only. The total amount of CO_2_ in each compartment was calculated by multiplying the total concentration by their volume.

In the current study, several parameters were assumed for average patients with ARDS. PBW was assumed as 85% of actual body weight, as approximated previously for critically ill patients.^24^ Blood hematocrit was fixed at 30%, and arterial blood base excess was assumed to be −3 mEq/L, a value intermediate between those reported in the literature for ARDS patients.^25^, ^26^ Cardiac output was assumed to be proportional to body surface area and was calculated in units of L/min using the relationship developed from The Strong Heart Study of 0.251 × (kg PBW)^0.67^.^27^ Certain parameters of the model were adjusted to agree with the current standard of care as defined by the average mechanical ventilation practices for treating ARDS patients from LUNG SAFE.^5^ Based on that report, the current standard of care prescription for treating average ARDS patients was a ventilation frequency of 20.8/min, a tidal volume of 7.6 mL/kg PBW, and a median fraction of inspired O_2_ of 0.6. Using the reported average partial pressure of O_2_ to fraction of inspired O_2_ ratio of 161 and average PaCO_2_ in arterial blood of 46.0 mm Hg,^5^ the pulmonary shunt fraction was estimated as 0.176, the O_2_ tissue uptake rate as 4.0 mL of O_2_ per kg PBW and a respiratory quotient as 0.966. Therefore, for patients with an actual body weight of 78 kg, as in LUNG SAFE,^5^ this work assumed the O_2_ tissue uptake rate was 265 mL of O_2_/min and the CO_2_ production rate was 256 mL of CO_2_/min. The pulmonary shunt fraction, O_2_ tissue uptake rate and respiratory quotient as reported above were fixed for all simulations in this study.

## 3 RESULTS

All calculated results assumed an actual patient body weight of 78 kg.

**Figure 2:**
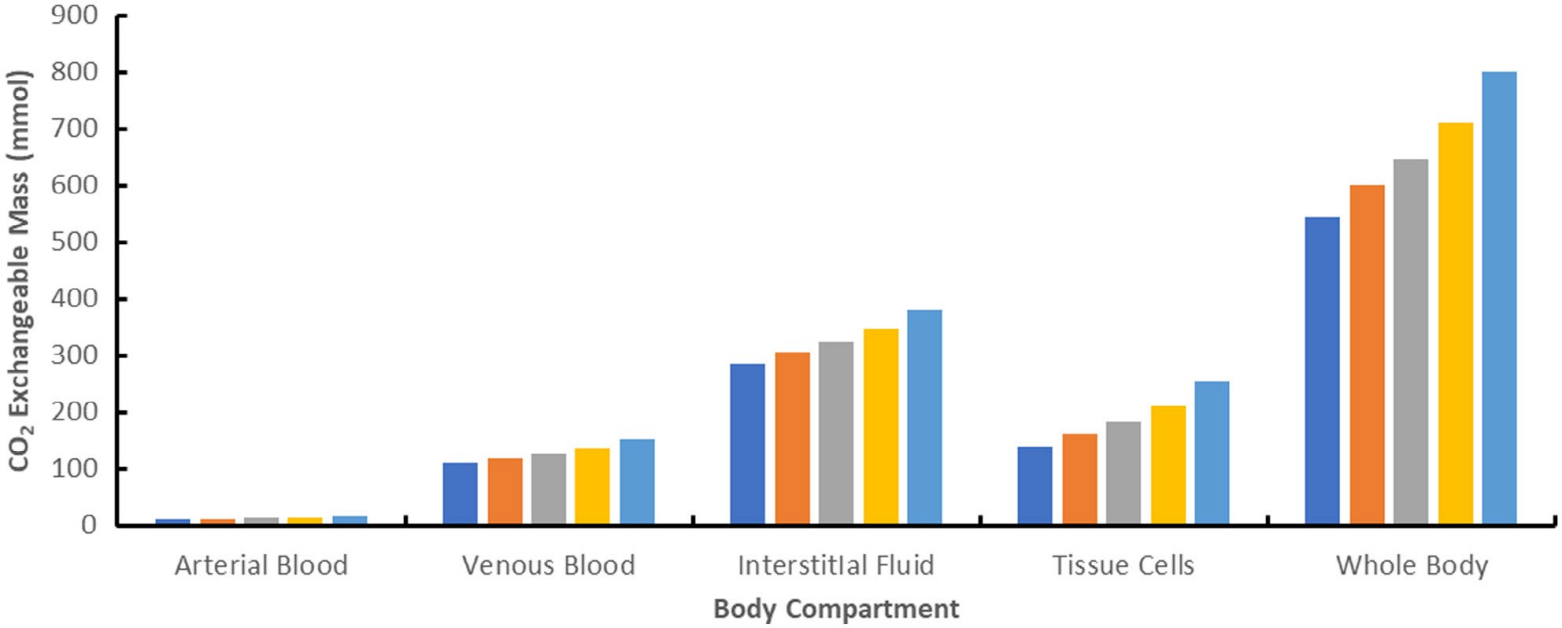
Effect of tidal volume on total body CO_2_ in the model compartments in the absence of the ECCO_2_R device. Results are shown at tidal volumes of 7.6 (dark blue bars), 6 (orange bars), 5 (gray bars), 4 (yellow bars), and 3 (light blue bars) mL/kg PBW [Color figure can be viewed at https://www.wileyonlinelibrary.com]

The effect of reductions in tidal volume during mechanical ventilation in ARDS patients on acid‐base chemistry was first evaluated in the absence of the ECCO_2_R device; Table 1 summarizes results from these initial model simulations. As calibrated by the initial assumed parameters, the partial pressure of arterial blood (PaCO_2_) at a tidal volume of 7.6 mL/kg PBW was identical to that reported in LUNG SAFE^5^ and the arterial blood (plasma) pH (pHa) was 7.32. At a tidal volume of 7.6 mL/kg PBW, total CO_2_ concentrations varied in the model compartments containing blood—the simulated total CO_2_ concentrations were 21.8 mM in arterial blood, 24.5 mM in venous blood, 27.3 mM in interstitial fluids, and 10.5 mM in tissue cells. When tidal volume decreased from 7.6 to 3 mL/kg PBW, PaCO_2_ increased and pHa decreased progressively as expected, and there was a maximal increase in total body mass of CO_2_ of approximately 50% when decreasing tidal volume from 7.6 to 3 mL/kg PBW. Note that the PaCO_2_ in venous blood (PvCO_2_) was calculated to be approximately 10 mm Hg higher than in arterial blood when the tidal volume was 7.6 mL/kg PBW, and this difference between PvCO_2_ and PaCO_2_ increased at lower tidal volumes. Figure 2 shows the effect of tidal volume on the total amount of CO_2_ in the various model compartments in the absence of the ECCO_2_R device. As expected, the total amount of CO_2_ in arterial blood was negligibly small and that in interstitial fluids was approximately one‐half of the total body CO_2_. There was a progressive increase in total mass of CO_2_ in each compartment as tidal volume was reduced.

**Table 1:** Effect of tidal volume on acid‐base blood chemistry and total body CO_2_ mass in the absence of the ECCO_2_R device (patient body weight of 78 kg with a mechanical ventilation frequency of 20.8/min)

Tidal Volume (mL/kg PBW)	PaCO_2_ (mm Hg)	pHa	PvCO_2_ (mm Hg)	pHv	Total Body CO_2_ (mmol)
7.6	46.0	7.32	55.5	7.28	544
6	58.0	7.25	69.1	7.21	600
5	69.3	7.20	82.0	7.16	647
4	86.3	7.13	101.0	7.09	710
3	114.5	7.04	132.5	7.00	801

PaCO_2_ denotes partial pressure of CO_2_ in arterial blood; pHa denotes the pH of arterial plasma; PvCO_2_ denotes partial pressure of CO_2_ in venous blood; pHv denotes the pH of venous plasma.

**Figure 3:**
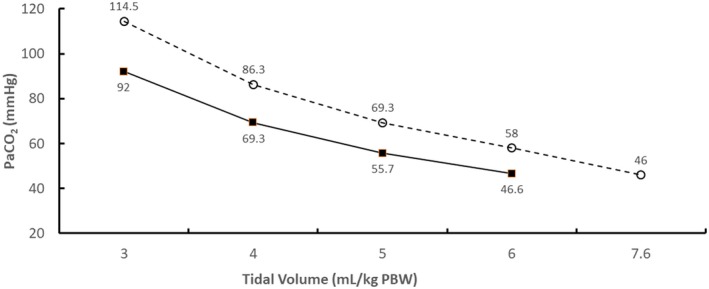
The effect of tidal volume and mechanical ventilation frequency on arterial partial pressure of CO_2_ (PaCO_2_) in the absence of the ECCO_2_R device. Results are shown for ventilation frequencies of 20.8/min (circles, dashed line) and 26/min (squares, solid line). Note that the results at a tidal volume of 7.6 mL/kg PBW were for a ventilation frequency of 20.8/min only

Figure 3 compares changes in PaCO_2_ when altering tidal volume and respiratory rate during mechanical ventilation in the absence of the ECCO_2_R device. Higher ventilation frequencies significantly reduced PaCO_2_ when tidal volume was reduced. The increase in respiratory rate from 20.8/min to 26/min also resulted in higher arterial pH by 0.07 units at each tidal volume (results not shown).

**Figure 4:**
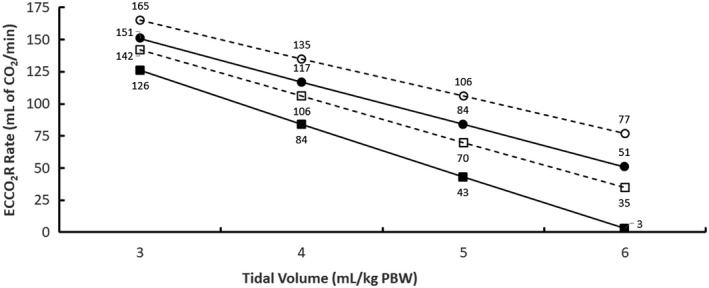
Calculated ECCO_2_R rate required to achieve a PaCO_2_ of 46 mm Hg (filled symbols, solid lines) and 40 mm Hg (open symbols, dashed lines) at various tidal volumes and mechanical ventilation frequencies. Results are shown for ventilation frequencies of 20.8/min (circles) and 26/min (squares)

The ECCO_2_R rate from the extracorporeal device to achieve a PaCO_2_ of 46 mm Hg (mild permissive hypercapnia), considered as standard of care based on LUNG SAFE,^5^ and 40 mm Hg (normocapnia) is shown in Figure 4 at ventilation frequencies of 20.8 and 26/min and various tidal volumes. As tidal volume was reduced, the required ECCO_2_R rate increased. Increasing ventilation frequency from 20.8 to 26/min substantially decreased the required ECCO_2_R rate. ECCO_2_R rates required to achieve normocapnia were correspondingly higher and were achieved at a PvCO_2_ of 48.7 mm Hg.

## 4 DISCUSSION

The current standard of care for mechanical ventilation in ARDS patients was identified in LUNG SAFE as mild permissive hypercapnia, a PaCO_2_ of 46 mm Hg, pHa of 7.33, and moderately low tidal volumes. Further reductions in tidal volume would be accompanied by elevated levels of PaCO_2_ and reductions in arterial pH, but permissive hypercapnia with PaCO_2_ ≥50 mm Hg during the first 48 hours after initiating mechanical ventilation is associated with an increased risk of intensive care unit mortality in ARDS patients.^28^ To ameliorate the unphysiological effects of hypercapnia, it has been established clinically that the use of ECCO_2_R results in variable reductions in PaCO_2_ within a few hours^29^; this is expected depending on the characteristics of the ECCO_2_R device used, as well as other clinical variables. As noted however by others,^29^ previous clinical studies have found it difficult to quantify the specific contribution of the ECCO_2_R device to the reduction in PaCO_2_. The approach taken in the current study was to quantify the relationship between the ECCO_2_R rate and changes in PaCO_2_ and other components of acid‐base chemistry in ARDS patients undergoing mechanical ventilation using a comprehensive biochemical and physiological simulation model.

This theoretical effort extends a previously published mathematical model of CO_2_ and O_2_ chemistry and whole‐body storage and transport^19^, ^21^ to provide estimates of the ECCO_2_R rate requirements for mechanically ventilated ARDS patients treated with low tidal volumes. Others^30^ have recently developed an alternative mathematical model to predict changes in PaCO_2_ as a function of treatment time and the blood flow rate for one specific ECCO_2_R device and compared their model predictions with empirical data from preclinical studies in a porcine model. The current mathematical model is comparable to this latter model in overall design but differs in three major ways. First, the current model only predicts the relationship between acid‐base chemistry and the ECCO_2_R rate; thus, the current approach is general and not dependent on the performance characteristics of a specific device. In practical clinical applications, the predictions from the current model may require supplementary data relating the ECCO_2_R rate to the blood flow rate for a specific device.^13^ Second, the current model is limited by the assumption of steady state conditions. The previous model^30^ was developed to explain time‐dependent changes in PaCO_2_ after abrupt, transient changes in the ECCO_2_R rate as well as time‐dependent changes in temperature and metabolic rate during the preclinical experiments. Those time‐dependent model elements were necessary to explain the data from their porcine experiments^30^ but are not relevant to the approximate steady state conditions when treating mechanically ventilated ARDS patients over the period of hours or days in the intensive care unit. Third, the current model provides a comprehensive description of CO_2_, O_2_, and acid‐base chemistry; thus, it is more general and applicable to a greater variety of clinical conditions. We believe our model and the previous model^30^ are complementary and applicable to different study objectives.

The structure of the mathematical model used in current study is however relatively simple from a physiological perspective. For example, we used a simple model of CO_2_ and O_2_ exchange in the lung that is unlikely to accurately represent the pathophysiological conditions in ARDS patients such as ventilation/perfusion inequalities.^31^ More extensive pulmonary gas exchange models^32^ can readily be incorporated into the current model if these predictions are empirically demonstrated to be quantitatively inaccurate. In addition, we have only considered a single tissue store of CO_2_ in muscle cells; thus, the estimate of tissue cell volume and mass of CO_2_ stored in tissues is likely an underestimate (see Figure 2). It should however be noted that the magnitude of tissue volume does not significantly influence the calculated ECCO_2_R rate in the current model as steady state conditions have been assumed. Moreover, specific phenomenon that might reduce device efficiency in vivo such as recirculation^13^ have also not been included in the current model. Our model is comparable in overall complexity to that recently described for extracorporeal membrane oxygenation.^33^

The current model demonstrates the strong dependence of PaCO_2_ and other components of acid‐base chemistry on respiratory rate during mechanical ventilation, both with and without ECCO_2_R. The use of high‐frequency ventilation was not evaluated in this study as it has been shown to not reduce, and may even increase, in‐hospital mortality,^34^ and such high‐frequency settings are not recommended for routine use in ARDS patients.^1^, ^3^ Although there is both theoretical^35^ and empirical^36^, ^37^, ^38^ evidence that low respiratory rates during mechanical ventilation may reduce VILI, it should be emphasized that the low tidal volume patient group in the ARDS Network trial^39^ was ventilated at respiratory rates of approximately 30/min and achieved low mortality rates. The higher ventilation frequencies considered in the current study were below these limits.

A major limitation of this work is the lack of empirical confirmation of the accuracy of the model predictions, partially because there are few clinical studies to date that have measured both ECCO_2_R rates and changes in acid‐base chemistry in ARDS patients. Nevertheless, other data in the literature do not contradict the results from the current work. For example, Winiszewski et al.^12^ reported that mechanically ventilated ARDS patients treated using a tidal volume of 5.3 mL/kg PBW and a respiratory rate of 26/min achieved a baseline PaCO_2_ of 50 mm Hg and a pH of 7.31; the former level in arterial blood is similar to that in Figure 3 at the same respiratory rate with a tidal volume of 6 mL/kg PBW (PaCO_2_ of 46.6 mm Hg). ECCO_2_R rates were not however reported from that study. In addition, Schmidt et al.^10^ reported that ARDS patients treated using a tidal volume of 6.1 mL/kg PBW and a ventilation frequency of 26/min achieved a baseline PaCO_2_ of 43 mm Hg and a pH of 7.39; again, the former level in arterial blood is similar to that in Figure 3 at the same ventilation frequency. In that study, patients who had tidal volumes reduced to 3.98 mL/kg PBW and received ECCO_2_R at a rate of 51 mL of CO_2_/min achieved a PaCO_2_ of 53 mm Hg. The calculated predictions in Figure 4 suggest that those patients could have achieved a lower PaCO_2_ of 46 mm Hg if the ECCO_2_R was increased to 84 mL of CO_2_/min.

As this study was being finalized to first submit for publication, the results from the SUPERNOVA trial were published.^14^ That prospective, multicenter, international phase 2 study of 95 mechanically ventilated ARDS patients demonstrated that it is feasible to use veno‐venous ECCO_2_R to allow reductions in tidal volume from 6 to 4 mL/kg PBW without a 20% increase in PaCO_2_ in approximately 80% of patients. Overall, reductions in tidal volume to 4 mL/kg PBW with simultaneous use of ECCO_2_R maintained PaCO_2_ at 46.7‐48.0 mm Hg with ventilation frequencies of 23.5‐27.4/min during the first 24 hours of treatment. A shortcoming of this trial was the inability to measure clearance and total amount of CO_2_ removal by ECCO_2_R as originally planned as a secondary endpoint of that trial.^14^ We can however use the mathematical predictions in Figure 4 to estimate the ECCO_2_R rate required to achieve approximately these same conditions (4 mL/kg PBW and ventilation frequency of 26/min) as 84 mL of CO_2_/min at a PvCO_2_ of 55.6 mm Hg (neglecting the calculated ECCO_2_R rate of 3 mL of CO_2_/min at a tidal volume of 6 mL/kg PBW). Obviously, higher ECCO_2_R rates would be required to achieve reductions in tidal volumes to 4 mL/kg PBW in closer to 100% of patients.

Additional limitations to this work include the following. First, the PBW could only be estimated from the actual body weight because the data necessary for calculating PBW in the LUNG SAFE publication were not reported. Second, model predictions were only reported for patients with an actual body weight of 78 kg. Because larger patients have higher rates of CO_2_ production, higher ECCO_2_R rates will likely be required. Most relevant parameters in the current model have been scaled to patient body weight; thus, a simple approach to extrapolate the reported required ECCO_2_R rates to other patients would be by body weight. Preliminary calculations from the current model have theoretically confirmed this hypothesis. Third, several other biochemical data were not reported in the LUNG SAFE publication, such as arterial bicarbonate concentration or base excess; those values could only be estimated based on values previously reported in the literature.

## 5 CONCLUSIONS

The current mathematical model predicts that ECCO_2_R rates resulting in clinically desirable PaCO_2_ levels at tidal volumes of 5‐6 mL/kg PBW can likely be achieved in mechanically ventilated ARDS patients with current technologies; use of ultraprotective tidal volumes (3‐4 mL/kg PBW) may be challenging unless higher mechanical ventilation frequencies are used. The recently reported secondary analysis from the SUPERNOVA trial that lower CO_2_ extraction devices required high mechanical ventilation frequencies to achieve clinically acceptable PaCO_2_ levels at ultraprotective tidal volumes^15^ supports the theoretical predictions in this work.

## CONFLICT OF INTEREST

JKL is a consultant to Baxter International and NxStage Medical Inc. (now Fresenius Medical Care). JG, DP, and KH are full‐time employees of Baxter International with ownership interests.

## AUTHOR CONTRIBUTIONS

JKL designed the study, developed the mathematical model, wrote the computer program, and wrote the first draft of the manuscript. DP provide advice on the development of the mathematical model. JG and KH provided clinical input into the study design. DP, JG, and KH also reviewed the manuscript and provided valuable input to the revisions of the manuscript. All authors read and approved the final manuscript.

## APPENDIX 1

Figure 1 shows a schematic of the whole‐body CO_2_ transport model used in this study. The model structure is identical to that proposed previously by others^19^ except to include an extracorporeal CO_2_ removal device into the venous circulation with a rate equal to JCO2. Only the four blood‐containing compartments (lung capillary blood exchanging gas with alveoli, arterial blood, venous blood, and post‐ECCO_2_R venous blood) from the six‐compartment model were used to formulate CO_2_ mass balance relationships. The transport of CO_2_ between the lung and the environment can be described by the following equations at steady state (following along the lines described by others^19^)

(A1) $${\dot{V}}_{A}=f\times \left[V_{T}-V_{D}\right]$$

(A2) $$\dot{V}\text{CO}2-\text{JCO}2={\dot{V}}_{A}\times \frac{P_{A}{\text{CO}}_{2}}{P_{B}-P_{H2O}}$$

where ${\dot{V}}_{A}$ denotes the volume of gas flowing into the alveoli per unit time, f denotes the respiratory frequency, V_T_ denotes the tidal volume, and V_D_ denotes the dead space volume. The net rate of CO_2_ exchange in the lung at steady state is equal to the tissue CO_2_ production rate ($\dot{V}$CO2) minus the extracorporeal CO_2_ removal rate by the ECCO_2_R device (JCO2) and can be related to the partial pressure of CO_2_ in the alveoli (P_A_CO_2_), the barometric pressure (P_B_) assumed equal to 760 mm Hg (101.3 kPa), and the pressure of saturated water (P_H2O_) assumed equal to 47 mm Hg (6.3 kPa) as shown in equation (A2). It should be noted that the fractional concentration of CO_2_ in inspired air is assumed to be zero in equation (A2). If it is also assumed that the PaCO_2_ in alveoli and lung capillary blood equilibrate, then equation (A2) can be rearranged to calculate the PaCO_2_ in lung capillary blood which can be used to calculate the total CO_2_ concentration in lung capillary blood (tCO_2,l_) from the acid‐base chemistry model described previously by others.^21^

The total CO_2_ concentration in arterial blood (tCO_2,a_) is a flow‐weighted average of that in lung capillary CO_2_ and in post‐ECCO_2_R venous blood (tCO_2,pv_) as given by

(A3) $${\text{tCO}}_{2,a}=\left(1-s\right)\times {\text{tCO}}_{2,l}+s\times {\text{tCO}}_{2,\text{pv}}$$

where s denotes the pulmonary shunt fraction. CO_2_ mass balance at steady state in the alveoli of the lung also requires

(A4) $$\dot{V}\text{CO}2-\text{JCO}2=Q\left(1-s\right)\left[{\text{tCO}}_{2,\text{pv}}-{\text{tCO}}_{2,l}\right]$$

where Q denotes cardiac output. Equations (A3) and (A4) can be combined to solve for tCO_2,a_ as

(A5) $${\text{tCO}}_{2,a}={\text{tCO}}_{2,l}+\frac{s\times \left[\dot{V}\text{CO}2-\text{JCO}2\right]}{Q\times \left(1-s\right)}$$

and then the total CO_2_ concentration in venous blood (tCO_2,v_) and tCO_2,pv_ can be sequentially calculated as

(A6) $${\text{tCO}}_{2,v}={\text{tCO}}_{2,a}+\frac{\dot{V}\text{CO}2}{Q}$$

(A7) $${\text{tCO}}_{2,\text{pv}}={\text{tCO}}_{2,v}-\frac{\text{JCO}2}{Q}$$

Note that equations (A5), (A6), and (A7) can be solved using the acid‐base chemistry model, as described by others,^21^ sequentially once the total CO_2_ concentration in lung capillary blood is known. Note also that solving the acid‐base chemistry model requires simultaneously solving O_2_ whole body transport relationships described by others^21^ (not shown here). All model equations were solved using Matlab (R2018a, The Mathworks Inc., Natick, MA, USA).
